# Fat-to-muscle ratio as a predictor for dyslipidaemia in transitional-age youth

**DOI:** 10.1186/s12944-022-01697-9

**Published:** 2022-09-19

**Authors:** Jia-Xing Zhang, Wen Li, Xiu-Juan Tao, Chen Chen, Qing-An Wang, Wan-Lu Liu, Chan Yang, Kai-Rong Wang, Jiang-Wei Qiu, Yi Zhao, Yu-Hong Zhang

**Affiliations:** 1 School of Public Health and Management, Ningxia Medical University, Yinchuan, Ningxia People’s Republic of China; 2 Department of Hospital Infection Management, People’s Hospital of Ningxia Hui Autonomous Region, Yinchuan, Ningxia People’s Republic of China; 3 Key Laboratory of Environmental Factors and Chronic Disease Control, Ningxia Medical University, Yinchuan, Ningxia People’s Republic of China

**Keywords:** Dyslipidaemia, Muscle, Fat-to-muscle ratio, Transitional-age youth

## Abstract

**Background:**

Although dyslipidaemia may have a crucial impact on cardiovascular health in adults, there is a lack of specific data in transitional-age youth. Therefore, this study attempted to evaluate the association of dyslipidaemia with fat-to-muscle ratio (FMR), and establish FMR thresholds for diagnosing dyslipidaemia in transitional-age youth.

**Methods:**

One thousand six hundred sixty individuals aged 16 to 24 years from the baseline of a subcohort in the Northwest China Natural Population Cohort: Ningxia Project were analysed. Anthropometric characteristics were gauged by a bioelectrical impedance analyser, and dyslipidaemia components were measured using a Beckman AU480 chemistry analyser. Additionally, this study used logistic regression to estimate the risk of dyslipidaemia based on FMR quintiles, and calculate the gender-specific ideal cut-off values of dyslipidaemia and its components by the receiver operating characteristic (ROC) curve.

**Results:**

Of the 1660 participants, aged 19.06 ± 1.14 years, 558 males and 1102 females. The prevalence of dyslipidaemia was 13.4% and was significantly associated with FMR quintiles among all participants (*P* < 0.05). The ideal values of FMR in diagnosing dyslipidaemia were 0.2224 for males and 0.4809 for females, while males had a higher AUC than females (0.7118 vs. 0.6656). Meanwhile, high FMR values were significantly associated with adverse outcomes of dyslipidaemia, hypercholesterolemia and hypertriglyceridaemia (*P* < 0.05).

**Conclusions:**

The FMR was positively correlated with the prevalence of dyslipidaemia. The FMR can be used as an effective body composition index for diagnosing dyslipidaemia, especially in males, and preventive strategies should be initiated in transitional-age youth to decrease obesity-related dyslipidaemia.

## Background

Obesity, a crucial risk factor for chronic diseases, is progressively becoming a global health issue [1, 2], with its prevalence increasing dramatically worldwide [3]. Obesity is associated not only with cardiovascular diseases in children [4] but also with vascular dysfunction and hormonal changes, leading to hypertension, dyslipidaemia and potential cardiovascular events in transitional-aged youth [5]. The transitional age period during youth is an important stage from adolescence to adulthood, ranging from the ages of 16 to 24 years [6]. Additionally, studies have demonstrated that the vast majority of individuals experience significant weight gain between the ages of 18 and 30 [7]. Thus, the incidence of cardiovascular diseases will increase in the future, which will lead to a global increase in deaths [8].

Previous investigations have proven dyslipidaemia to be associated with adult atherosclerosis [9] and regarded it as an effective indicator for predicting future cardiovascular events [10]. In addition, due to the close correlation between obesity and dyslipidaemia, body mass index (BMI), waist circumference (WC) and other obesity-related indicators have already been used to assess dyslipidaemia, metabolic syndrome and obesity-related cardiovascular disease risks [11–14]. However, BMI cannot accurately reflect muscle and fat content, and WC cannot be used to reflect visceral fat [15, 16]. Moreover, several studies have also suggested using different body composition measures to assess future cardiovascular disease risks [17–19]. Notably, fat and muscle mass may be major contributors to metabolic syndrome and cardiovascular diseases [20, 21], and fat mass (FM) is even regarded as an effective indicator to predict metabolic syndrome [18]. Fat accumulation and skeletal muscle attenuation occur simultaneously and are often expressed as the fat-to-muscle ratio (FMR), a substitutable measure for evaluating the proportion of fat and muscle [22].

Recently, the FMR, as a novel anthropometric indicator, has been used to assess dyslipidaemia [23], metabolic syndrome [24] and coronary artery disease [25] in healthy adults. Although the FMR is also considered an indicator of metabolic syndrome in Chinese Han and Buyi populations aged 20 to 80 years [26], there is no agreement on the definition of dyslipidaemia in the context of FMR. Furthermore, the prediction of adult dyslipidaemia has been improved through a variety of measurement methods, but there is a lack of specific data in transitional-age youth. Moreover, current guidelines recommend screening young people for dyslipidaemia [27, 28]. Accordingly, this study hypothesized that the FMR is a feasible diagnostic index for dyslipidaemia in transitional-age youth, explored the association of dyslipidaemia with the FMR, and established the FMR threshold for the diagnosis of dyslipidaemia.

## Methods

### Study participants

This study is the baseline of a subcohort in the Northwest China Natural Population Cohort: Ningxia Project (CNC-NX), conducted with 1720 transitional-age youth aged 16 to 24 in September 2018. At enrolment, general questionnaires were administered to all participants; subsequently, a battery of anthropometric measurements was completed, and blood samples were used to collect data on biological indicators.

**Fig. 1:**
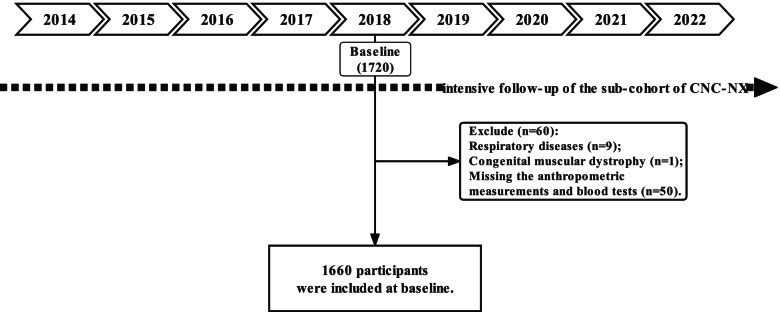
An outline of the procedure for choosing the study population

In this prospective study, participants who had studied in the survey area for 3 years or more were included. Participants with poor health status or diseases potentially affecting their body composition were excluded, such as respiratory diseases (*n* = 9) and congenital muscular dystrophy (*n* = 1). Simultaneously, participants who had missing anthropometric measurements and blood tests (*n* = 50) were also excluded from the final study analysis (Fig. 1). Ultimately, 1660 eligible participants were included.

The institutional ethics committees at Ningxia Medical University gave their approval for this study (Ethics ID 2018–012, 2020–689), and at the start of the survey, each participant signed a consent form after receiving full information.

### Data collection

Trained investigators collected information and baseline data in September 2018, and all the following measures were recorded for each participant.

#### Demographic data

Following the signing of the informed consent form, participants were invited to fill in a face-to-face questionnaire that included demographic characteristics, including age, sex, marital status, education level, and health conditions, such as lifestyle and behavioural factors, medical history and menstrual history [29]. The information on smoking and alcohol drinking status was defined as smoking ≥ 1 cigarette daily sustained for ≥ 6 months and drinking ≥ 1 time per week sustained for ≥ 6 months, respectively [30]. Education level was divided into two categories: junior college education level (more than or equal to a senior high school) and undergraduate education level. Physical activity (PA) was assessed using the International Physical Activity Questionnaire [31], and graded as low, moderate, or high by the World Health Organization (WHO) guidelines [32].

#### Anthropometric measurements

The participants fasted for at least 12 h, avoided alcohol, wore light clothing with no shoes, and were measured while standing. Weight and height were measured twice, with averages to the nearest 0.1 kg and 0.1 cm, respectively. Body composition was measured by trained personnel using a single frequency, eight-electrode bioelectrical impedance analyser (BIA) (InBody 370, Seoul, Korea) in accordance with the recommended procedures. Several anthropometric measurements were recorded for the participants, including their FM, total body soft lean mass, skeletal muscle mass (SMM), and other anthropometric factors.

#### Experimental measurements

Participants fasted the night before their venous blood was drawn. Using the Beckman AU480 chemistry analyser, fasting blood glucose (FBG), total cholesterol (TC), triglycerides (TGs), high-density lipoprotein cholesterol (HDL-C), and low-density lipoprotein cholesterol (LDL-C) were measured.

### Definition of covariates

Whole-body skeletal muscle mass can be replaced with appendicular skeletal muscle mass (ASM), determined by adding the limb muscle mass together [33]. Calculating the percentage of skeletal muscle mass (ASM %) requires dividing ASM by body weight [34]. BMI was calculated as weight/height^2^ (kg/m^2^) [35]. FM was divided by the total body soft lean mass to determine the FMR, which was then divided into quintiles (Q1-Q5) from lowest to highest values. The ranges of FMR across quintiles were < 0.3173, 0.3173- < 0.3772, 0.3772- < 0.4324, 0.4324- < 0.5094, ≥ 0.5094 for female participants; and < 0.1314, 0.1314- < 0.1712, 0.1712- < 0.2352, 0.2352- < 0.3262, ≥ 0.3262 for male participants.

### Dyslipidaemia

Dyslipidaemia was defined based on any one of the following characteristics: TC ≥ 6.20 $\mathrm{mmol}/\mathrm{L}$ (240 mg/dl), TGs ≥ 2.30 $\mathrm{mmol}/\mathrm{L}$ (200 mg/dl), LDL-C ≥ 4.10 $\mathrm{mmol}/\mathrm{L}$ (160 mg/dl), HDL-C < 1.00 $\mathrm{mmol}/\mathrm{L}$ (40 mg/dl) or receiving drug treatment to improve blood lipid levels [36]. In addition, hypercholesterolemia was defined as TC ≥ 6.20 $\mathrm{mmol}/\mathrm{L}$ (240 mg/dl) and hypertriglyceridaemia as TGs ≥ 2.30 $\mathrm{mmol}/\mathrm{L}$ (200 mg/dl).

### Statistical methods

R 4.0.0 software was used to statistically analyse the research datasets. For continuous variables, the mean and standard deviation (SD) were used as representations. The number of cases and the rate were used to express categorical variables. After determining normality and variance homogeneity with the Kolmogorov–Smirnov test and Levene's test, Student’s T and χ^2^ tests were used to compare general characteristics by sex, and the T-test was utilized to compare anthropometric parameters according to dyslipidaemia and nondyslipidaemia. ANOVA and χ^2^ tests were used to compare dyslipidaemia among the FMR quintiles based on sex. Additionally, this study used logistic regression to estimate the risk of dyslipidaemia based on FMR quintiles, and the statistically significant variables from the univariate analysis results were considered in the multivariable model. While the variables with a variance inflation factor (VIF) < 5 were chosen and included in the final adjustment model, multicollinearity diagnosis was also performed on the included variables. Finally, the odds ratio (OR) and 95% confidence interval (CI) were computed after taking age, smoking, drinking, physical activity, education level, and ethnicity into account.

To establish the cut-off values for the FMR, the receiver operating characteristic (ROC) curve was used, with a standard for identifying dyslipidaemia as the ROC curve that is most closely related to (0, 1). Moreover, the optimal cut-off FMR value was obtained based on a maximized Youden’s index, and the sensitivity, specificity and area under the ROC curve (AUC) were also examined. Following participant division was founded on the cut-off FMR value, the Student’s T test and the χ^2^ test were utilized to compare the dyslipidaemia risk levels among the groups. Every statistical test used two sides, and *P* < 0.05 indicates statistically significant.

## Results

### General characteristics

Of the 1660 participants, aged 19.06 ± 1.14 years, 558 males and 1102 females. Regarding anthropometric measurements, men had higher weight, height, BMI, WC, ASM, ASM%, and soft lean mass but lower FM and FMR values than women (*P* < 0.001). Regarding the laboratory measurements, men had higher levels of TC, TGs, and FBG and an even higher prevalence of dyslipidaemia than women (*P* < 0.05). Furthermore, men also had higher levels of drinking and smoking consumption than women (*P* < 0.001). As shown in Table 1.

**Table 1:** Characteristics of the subjects

Variables	Total (*n* = 1660)	Males (*n* = 558)	Females (*n* = 1102)	*t*/*χ*^*2*^	*P* value
Age (years)	19.06 ± 1.14	19.17 ± 1.24	19.00 ± 1.09	2.839	0.005
Education level (n, %)					
Junior College	552 (33.3)	93 (16.7)	459 (41.7)	104.184	< 0.001
Undergraduate	1108 (66.7)	465 (83.3)	643 (58.3)		
Weight (kg)	56.73 ± 10.87	64.32 ± 11.48	52.89 ± 8.21	23.316	< 0.001
Height (cm)	166.13 ± 7.91	174.29 ± 5.73	162.00 ± 5.18	44.025	< 0.001
BMI (kg/m^2^)	20.48 ± 3.12	21.15 ± 3.50	20.13 ± 2.85	6.367	< 0.001
WC (cm)	74.67 ± 8.39	77.46 ± 9.81	73.25 ± 7.17	9.935	< 0.001
ASM (kg)	18.30 ± 4.29	23.30 ± 2.89	15.76 ± 2.09	60.631	< 0.001
ASM %	0.32 ± 0.04	0.37 ± 0.04	0.30 ± 0.03	42.029	< 0.001
FM (kg)	13.92 ± 6.05	11.72 ± 6.78	15.04 ± 5.30	-10.941	< 0.001
Soft Lean Mass	40.36 ± 8.10	49.71 ± 5.95	35.63 ± 3.78	58.615	< 0.001
FMR	0.36 ± 0.15	0.23 ± 0.12	0.42 ± 0.12	-29.479	< 0.001
Cholesterol ($\mathrm{mmol}/\mathrm{L}$)	4.56 ± 1.02	4.66 ± 1.01	4.50 ± 1.01	3.162	0.002
HDL-C ($\mathrm{mmol}/\mathrm{L}$)	1.38 ± 0.34	1.31 ± 0.32	1.42 ± 0.35	-6.336	< 0.001
LDL-C ($\mathrm{mmol}/\mathrm{L}$)	1.00 ± 0.33	0.98 ± 0.30	1.00 ± 0.34	-1.213	0.225
Triglycerides ($\mathrm{mmol}/\mathrm{L}$)	0.95 ± 0.50	1.08 ± 0.61	0.89 ± 0.42	7.279	< 0.001
Glucose ($\mathrm{mmol}/\mathrm{L}$)	4.68 ± 0.61	4.73 ± 0.63	4.66 ± 0.60	2.210	< 0.001
Dyslipidaemia (n, %)	223 (13.4)	102 (18.3)	121 (11.0)	16.973	< 0.001
Smoking (n, %)	69 (4.2)	68 (12.2)	1 (0.1)	135.931	< 0.001
Alcohol intake (n, %)	33 (2.0)	26 (4.7)	7 (0.6)	30.753	< 0.001
Physical activity (n, %)					
Low	365 (22.0)	89 (15.9)	276 (25.0)	48.064	< 0.001
Medium	567 (34.1)	159 (28.5)	408 (37.1)		
High	728 (43.9)	310 (55.6)	418 (37.9)		

*P* < 0.05 was considered statistically significant

### Correlations of dyslipidaemia with general characteristics

The entire participant pool was split into two groups based on dyslipidaemia status (dyslipidaemia and nondyslipidaemia); as shown in Table 2, sex, educational background, WC, ASM, ASM%, FM, BMI, and the FMR were significantly correlated with dyslipidaemia (*P* < 0.05). Conversely, the mean age, smoking and alcohol intake status, and physical activity showed no significant differences between transitional-age youth with and without dyslipidaemia. Moreover, the FMR in transitional-age youth with dyslipidaemia was higher than that in those without dyslipidaemia (*P* < 0.05).

**Table 2:** Baseline characteristics among subjects by dyslipidaemia and nondyslipidaemia status

Variables	Dyslipidaemia		Nondyslipidaemia	*t*/*χ*^*2*^	*P* value
	(*n* = 223)		(*n* = 1437)		
Age (years)	19.07 ± 1.20		19.07 ± 1.14	0.066	0.947
Female (n, %)	121 (54.3)		981 (68.3)	16.973	< 0.001
Smoking (n, %)	14 (6.3)		55 (3.8)	2.977	0.084
Alcohol intake (n, %)	4 (1.8)		29 (2.0)	0.045	0.831
Education level (n, %)					
Junior College	35 (15.7)		517 (36.0)	35.780	< 0.001
Undergraduate	188 (84.3)		920 (64.0)		
WC (cm)	80.00 ± 10.86		73.84 ± 7.61	10.532	< 0.001
ASM (kg)	19.87 ± 4.79		18.05 ± 4.16	5.956	< 0.001
ASM %	0.31 ± 0.04		0.32 ± 0.04	-2.991	0.003
FM (kg)	17.03 ± 7.37		13.44 ± 5.66	8.417	< 0.001
BMI (kg/m^2^)	22.50 ± 3.84		20.16 ± 2.87	10.747	< 0.001
FMR	0.40 ± 0.17		0.35 ± 0.15	4.574	< 0.001
FBG ($\mathrm{mmol}/\mathrm{L}$)	4.71 ± 0.63		4.67 ± 0.61	0.862	0.389
Physical activity (n, %)					
Low	58 (26.0)		307 (21.4)	4.261	0.119
Medium	64 (28.7)		503 (35.0)		
High	101 (45.3)		627 (43.6)		

*P* < 0.05 was considered statistically significant

### Correlations between dyslipidaemia and FMR

Table 3 demonstrates the significant relationship among BMI, dyslipidaemia, and dyslipidaemia components, except HDL-C, and the FMR quintiles. The prevalence of dyslipidaemia increased with the FMR, even after adjustment for possible confounders, for both males and females (*P* < 0.001; Table 4). In comparison to Q1, the corrected ORs values of dyslipidaemia in FMR Q2, Q3, Q4, and Q5 were 1.57 (95% CI: 0.61–4.03), 2.22 (95% CI: 0.90–5.46), 3.29 (95% CI: 1.39–7.81), and 7.56 (95% CI: 3.29–17.38), respectively, for males and 0.74 (95% CI: 0.34–1.60), 1.26 (95% CI: 0.63–2.52), 1.45 (95% CI: 0.74–2.88) and 3.04 (95% CI: 1.63–5.67), respectively, for females.

**Table 3:** Obesity-related characteristics of the subjects according to FMR quintiles

Sex	Variables	Total	Q1	Q2	Q3	Q4	Q5	*F*/*χ*^*2*^	*P* value	Post hoc analyses
Men		(*n* = 558)	(*n* = 112)	(*n* = 112)	(*n* = 111)	(*n* = 112)	(*n* = 111)			
	FMR	0.23 ± 0.12	0.10 ± 0.02	0.15 ± 0.01	0.20 ± 0.02	0.28 ± 0.03	0.42 ± 0.09	962.114	< 0.001	Q1 < Q2 < Q3 < Q4 < Q5
	BMI (kg/m^2^)	21.15 ± 3.50	18.21 ± 1.53	19.16 ± 1.62	19.84 ± 1.97	22.67 ± 2.23	25.92 ± 2.88	248.660	< 0.001	Q1 < Q2 < Q3 < Q4 < Q5
	Dyslipidaemia (n, %)	102 (18.3)	8 (7.1)	12 (10.7)	17 (15.3)	23 (20.5)	42 (37.8)	43.049	< 0.001	Q1 < Q4 < Q5; Q2, Q3 < Q5
Dyslipidaemia components										
	HDL-C	1.31 ± 0.32	1.35 ± 0.30	1.33 ± 0.32	1.30 ± 0.27	1.30 ± 0.35	1.25 ± 0.33	1.532	0.192	
	LDL-C	0.98 ± 0.30	0.91 ± 0.28	0.90 ± 0.25	0.94 ± 0.30	1.03 ± 0.32	1.15 ± 0.27	14.574	< 0.001	Q1, Q2 < Q4 < Q5; Q3 < Q5
	TC	4.66 ± 1.01	4.40 ± 0.87	4.41 ± 0.93	4.46 ± 0.99	4.83 ± 1.06	5.22 ± 0.96	15.346	< 0.001	Q1, Q2 < Q4 < Q5; Q3 < Q5
	TGs	1.08 ± 0.61	0.85 ± 0.36	0.95 ± 0.55	1.03 ± 0.50	1.10 ± 0.57	1.47 ± 0.82	18.405	< 0.001	Q1 < Q3, Q4, Q5; Q1, Q2, Q3, Q4 < Q5
Women		(*n* = 1102)	(*n* = 221)	(*n* = 220)	(*n* = 221)	(*n* = 220)	(*n* = 220)			
	FMR	0.42 ± 0.12	0.27 ± 0.04	0.35 ± 0.02	0.40 ± 0.02	0.47 ± 0.02	0.61 ± 0.09	1660.967	< 0.001	Q1 < Q2 < Q3 < Q4 < Q5
	BMI (kg/m^2^)	20.13 ± 2.85	17.64 ± 1.36	18.79 ± 1.47	19.82 ± 1.56	20.69 ± 1.70	23.75 ± 3.14	309.240	< 0.001	Q1 < Q2 < Q3 < Q4 < Q5
	Dyslipidaemia (n, %)	121 (11.0)	16 (7.2)	15 (6.8)	21 (9.5)	25 (11.4)	44 (20.0)	25.901	< 0.001	Q1, Q2, Q3 < Q5
	Dyslipidaemia components									
	HDL-C	1.42 ± 0.35	1.41 ± 0.37	1.47 ± 0.39	1.41 ± 0.32	1.41 ± 0.35	1.39 ± 0.32	1.780	0.131	
	LDL-C	1.00 ± 0.34	0.93 ± 0.27	0.98 ± 0.37	1.00 ± 0.36	1.01 ± 0.35	1.08 ± 0.37	5.249	< 0.001	Q1 < Q5
	TC	4.48 ± 1.01	4.18 ± 0.86	4.33 ± 1.04	4.48 ± 0.92	4.53 ± 1.00	4.91 ± 1.07	17.303	< 0.001	Q1 < Q3, Q4; Q1, Q2, Q3, Q4 < Q5
	TGs	0.89 ± 0.42	0.82 ± 0.43	0.84 ± 0.30	0.87 ± 0.37	0.91 ± 0.42	1.02 ± 0.53	8.205	< 0.001	Q1, Q2, Q3 < Q5

*P* < 0.05 was considered statistically significant

**Table 4:** Logistic regression analysis of association between FMR and the risk of dyslipidaemia

Sex	Quintile of the FMR	Crude	Age adjusted	Multivariable ^a^
Men (*n* = 558)				
	Q1 (*n* = 112)	Reference	Reference	Reference
	Q2 (*n* = 112)	1.56 (0.61–3.98)	1.57 (0.61–4.00)	1.57 (0.61–4.03)
	Q3 (*n* = 111)	2.35 (0.97–5.70)	2.35 (0.97–5.71)	2.22 (0.90–5.46)
	Q4 (*n* = 112)	**3.36 (1.43–7.88)**	**3.38 (1.44–7.95)**	**3.29 (1.39–7.81)**
	Q5 (*n* = 111)	**7.91 (3.50–17.88)**	**7.94 (3.51–17.94)**	**7.56 (3.29–17.38)**
	*χ*^*2*^_trend_	43.049	43.315	51.445
	*P*_trend_	< 0.001	< 0.001	< 0.001
Women (*n* = 1102)				
	Q1 (*n* = 221)	Reference	Reference	Reference
	Q2 (*n* = 220)	0.94 (0.45–1.95)	0.92 (0.44–1.92)	0.74 (0.34–1.60)
	Q3 (*n* = 221)	1.35 (0.68–2.65)	1.33 (0.68–2.63)	1.26 (0.63–2.52)
	Q4 (*n* = 220)	1.64 (0.85–3.17)	1.62 (0.84–3.13)	1.45 (0.74–2.88)
	Q5 (*n* = 220)	**3.20 (1.75–5.88)**	**3.16 (1.72–5.81)**	**3.04 (1.63–5.67)**
	*χ*^*2*^_trend_	25.901	26.148	62.248
	*P*_trend_	< 0.001	< 0.001	< 0.001

^a^ Multivariable: age, smoking, drinking, physical activity, level of education, and ethnicity were taken into account

*P* < 0.05 was considered statistically significant

### The FMR cut-off value for dyslipidaemia and its components

**Fig. 2:**
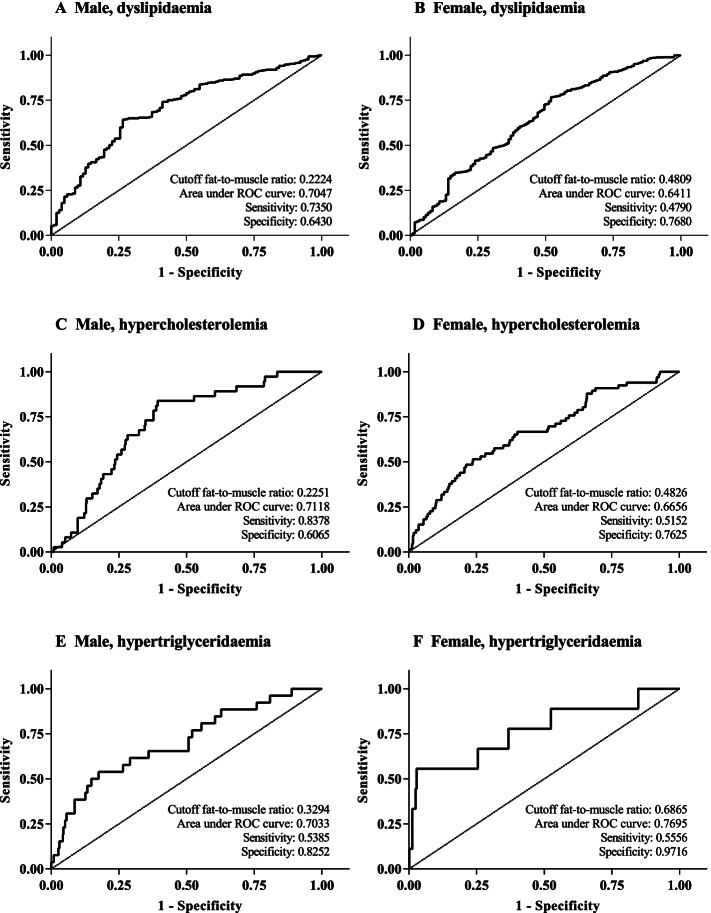
The sex-specific FMR cut-off points and ROC curves for identifying dyslipidaemia and its components. (**A**) Males, dyslipidaemia; (**B**) Females, dyslipidaemia. (**C**) Males, hypercholesterolemia; (**D**) Females, hypercholesterolemia. (**E**) Males, hypertriglyceridaemia; (**F**) Females, hypertriglyceridaemia

Figure 2 displays the gender-specific ROC curves for dyslipidaemia and its components. For detecting dyslipidaemia, the cut-off value of the FMR was 0.2224 for males and 0.4809 for females and specificity was lower in males than in females (0.6430 vs. 0.7680). The AUC and sensitivity were also higher in males than in females (0.7047, 0.7350 vs. 0.6411, 0.4790). Furthermore, additional secondary analyses were performed for the ability of the FMR to predict hypercholesterolemia and hypertriglyceridaemia. For predicting hypercholesterolemia, the cut-off ratio value, sensitivity and specificity were 0.2251, 0.8378, and 0.6065 in males and 0.4826, 0.5152, and 0.7625 in females, while males had a higher AUC than females (0.7118 vs. 0.6656). For predicting hypertriglyceridaemia, the cut-off ratio, AUC, sensitivity and specificity were lower in males than in females (0.3294, 0.7033, 0.5385, and 0.8252 vs. 0.6865, 0.7695, 0.5556, and 0.9716). The sex-specific cut-off point of FMR for identifying higher risks of dyslipidaemia indicates that those with elevated FMR are more likely to experience adverse outcomes from dyslipidaemia (*P* < 0.05; Table 5). Meanwhile, the multivariable-adjusted ORs of dyslipidaemia, hypercholesterolemia and hypertriglyceridaemia according to the sex-specific FMR cut-off level were significant (Table 6), which were 4.67 (95% CI: 2.85–7.63), 6.85 (95% CI: 2.77–16.96), and 2.41 (95% CI: 1.04–5.60), respectively, in men and 3.01 (95% CI: 2.07–4.49), 3.20 (95% CI: 1.91–5.38), and 4.60 (95% CI: 1.07–19.83), respectively, in women.

**Table 5:** Fat-to-muscle ratio detection thresholds based on sex

Characteristic	Males			Females		
	FMR < 0.2224	FMR ≥ 0.2224	*P* value	FMR < 0.4809	FMR ≥ 0.4809	*P* value
	(*n* = 319)	(*n* = 239)		(*n* = 816)	(*n* = 286)	
Anthropometric parameters						
Weight (kg)	57.68 ± 6.44	73.19 ± 10.72	< 0.001	50.13 ± 5.73	60.75 ± 9.10	< 0.001
BMI (kg/m^2^)	18.96 ± 1.77	24.08 ± 3.08	< 0.001	19.09 ± 1.84	23.12 ± 3.10	< 0.001
WC (cm)	70.92 ± 3.90	86.19 ± 8.42	< 0.001	70.52 ± 4.35	81.06 ± 7.87	< 0.001
Body fat mass (kg)	7.06 ± 2.06	17.94 ± 5.84	< 0.001	12.75 ± 2.92	21.56 ± 5.12	< 0.001
Blood lipid parameters						
TC ($\mathrm{mmol}/\mathrm{L}$)	4.40 ± 0.88	5.01 ± 1.08	< 0.001	4.35 ± 0.95	4.85 ± 1.08	< 0.001
TGs ($\mathrm{mmol}/\mathrm{L}$)	0.93 ± 0.48	1.27 ± 0.72	< 0.001	0.85 ± 0.38	1.02 ± 0.51	< 0.001
HDL-C ($\mathrm{mmol}/\mathrm{L}$)	1.33 ± 0.29	1.27 ± 0.34	0.030	1.43 ± 0.36	1.37 ± 0.33	0.004
LDL-C ($\mathrm{mmol}/\mathrm{L}$)	0.91 ± 0.27	1.08 ± 0.31	< 0.001	0.97 ± 0.33	1.08 ± 0.39	< 0.001
FBG ($\mathrm{mmol}/\mathrm{L}$)	4.72 ± 0.63	4.74 ± 0.64	0.722	4.64 ± 0.60	4.69 ± 0.60	0.263
Dyslipidaemia (n, %)	27 (8.5)	75 (31.4)	< 0.001	63 (7.7)	58 (20.3)	< 0.001

*P* < 0.05 was considered statistically significant

**Table 6:** Odds ratios for dyslipidaemia and its components according to the sex-specific FMR cut-off level

Sex	Outcome variables	Crude	Age adjusted	Multivariable ^a^
Men (*n* = 558)				
	Dyslipidaemia	4.95 (3.06–7.99)	4.97 (3.07–8.02)	4.67 (2.85–7.63)
	Hypercholesterolemia	7.78 (3.19–18.96)	7.79 (3.19–18.99)	6.85 (2.77–16.96)
	Hypertriglyceridaemia	2.64 (1.16–6.03)	2.64 (1.16–6.03)	2.41 (1.04–5.60)
Women (*n* = 1102)				
	Dyslipidaemia	3.04 (2.07–4.47)	3.03 (2.06–4.45)	3.01 (2.07–4.49)
	Hypercholesterolemia	3.31 (2.00–5.47)	3.23 (1.95–5.36)	3.20 (1.91–5.38)
	Hypertriglyceridaemia	5.81 (1.44–23.37)	5.70 (1.41–22.97)	4.60 (1.07–19.83)

^a^ Multivariable: age, smoking, drinking, physical activity, level of education, and ethnicity were taken into account

*P* < 0.05 was considered statistically significant

## Discussion

According to previous studies, in addition to a high BMI, which is often used as an effective indicator of obesity and cardiovascular disease risk across a wide population, some body composition measurements have been used to detect cardiovascular disease risk, which has been well reported in several previous studies [37, 38]. The proportion of visceral adipose to thigh muscle area was thought to be a suitable indicator of glycometabolism and insulin resistance in middle-aged women [39, 40]. The SMM, FM, and body fat percentage were linked to metabolic syndrome [17, 41, 42], and muscle strength was inversely correlated with the risk of cardiovascular diseases [43]. In addition, a loss of muscle mass can account for decreases in physical activity and the basal metabolic rate. Conversely, visceral obesity, sarcopenic obesity and high FMI are favourably correlated with metabolic syndrome and cardiovascular diseases [44, 45]. However, the danger of cardiovascular diseases cannot currently be assessed simultaneously by a comprehensive predictor, although various types of body composition indicators have been used to predict the validity of metabolic dysfunction. Compared to other body composition indices, the FMR is thought of as a new-type predictor for metabolic syndrome [22] and cardiovascular disease risk [40] in recent years.

Furthermore, dyslipidaemia in childhood, adolescence, and even during the transitional period of youth may have a crucial impact on cardiovascular health in adulthood. Additionally, the connection between accumulated fat and dyslipidaemia has been revealed in numerous studies, and the non-high-density lipoprotein cholesterol and obesity indices are related and considered useful screening tools for atherosclerotic cardiovascular disease risk [46, 47]. Similarly, the risk of dyslipidaemia has also been shown to be significantly increased by low skeletal muscle mass [48]. However, the passage from adolescence to adulthood seems to be marked by significant changes in lifestyle that affect the emergence of obesity. Furthermore, the quality of life of patients was positively impacted by conventional lipid-lowering drugs [49, 50], but patients with dyslipidaemia may have side effects (such as muscle symptoms) during treatment [51], which will affect their muscle health and lead to the further deterioration of their physical condition. Therefore, this research is more concerned about the connection between dyslipidaemia and changes in the FMR in transitional-age youth.

### Comparisons with other studies and what does the current work add to the existing knowledge

The present study, which is the baseline of a subcohort from the Northwest China Natural Population Cohort: Ningxia Project (CNC-NX), revealed a positive correlation between the prevalence of dyslipidaemia and a high FMR value. Additionally, the FMR served as an effective predictor for diagnosing dyslipidaemia, and the sensitivity of the cut-off FMR value was high in males, while the specificity of the cut-off FMR value was high in females. Moreover, there has never been a study with transitional-age youth to examine the relationships between the FMR and dyslipidaemia and to establish thresholds to facilitate the diagnosis of a high risk of dyslipidaemia that we are aware of.

According to prior research, FMR is a more accurate potential predictor of cardiovascular disease risk assessment than other individual components and has been applied in clinical practice [52, 53]. Several studies measured body composition by BIA and developed the ideal FMR cut-off value for metabolic syndrome detection [22]. Similarly, this study also found the association of the highest FMR value with dyslipidaemia at baseline, and the ideal values of FMR in diagnosing dyslipidaemia were 0.2224 for males and 0.4809 for females, which supported the hypothesis that the FMR is a feasible predictive index of dyslipidaemia in transitional-age youth. The potential mechanism is that adipocytes and macrophages associated with adipose tissue secrete more pro-inflammatory adipokines as body fat accumulation, including tumor necrosis factor-α and serum amyloid A, which may lead to a discrepancy between pro- and anti-inflammatory adipokines and promote dyslipidaemia [54, 55]. Meanwhile, skeletal muscle is regarded as an essential insulin-responsive endocrine organ, and muscle loss worsens glycemic control and insulin sensitivity, which may facilitate the onset of dyslipidaemia [56, 57]. Thus, the simultaneous occurrence of fat accumulation and skeletal muscle reduction can cause muscle inflammation and adversely affect myocyte metabolism, resulting in insulin resistance and promoting dyslipidaemia [58, 59].

Based on an earlier study, which observed that the FMR increased with age from 35 to 74 years [29], while this phenomenon may also have occurred in transitional-age youth in the current study. Therefore, preventive strategies can be initiated in transitional-age youth to decrease cardiovascular risk factors in adulthood, thereby reducing the morbidity and mortality of future heart diseases. Importantly, to clarify the exact mechanism between the FMR and the risks of dyslipidaemia, future longitudinal research and further work are particularly needed.

### Study strengths and limitations

Many advantages come from this study: potential confounding elements such as socioeconomic status and lifestyle were taken into account when conducting the analyses for this study. In addition, this study also determined the difference in the FMR in predicting dyslipidaemia according to sex. In addition, the current study has some constraints that should be considered. First, BIA, a trustworthy and practical technique, was used in place of dual-energy X-ray absorptiometry, the industry standard for human body composition detection [60]. However, this research used unified measurement methods at baseline and follow-up to avoid errors as much as possible. Second, the analysis data were from the baseline data of a cohort study and a relatively small sample of a transitional-age youth population, so the application range of the cut-off FMR values is limited.

## Conclusions

Many guidelines recommend early screening for dyslipidaemia before adulthood. This study demonstrated that the FMR serves as a practical predictor for dyslipidaemia, especially in males. Therefore, keeping a relatively low FMR is beneficial for preventing dyslipidaemia in transitional-age youth. Meanwhile, FMR should be taken into account in lipid management in clinical practice and preventive strategies should be initiated in transitional-age youth to decrease obesity-related dyslipidaemia.

## Data Availability

The datasets used and/or analysed during the current study are available from the corresponding author on reasonable request.

## Abbreviations

FMR: Fat-to-muscle Ratio
BMI: Body mass index
WC: Waist circumference
FM: Fat mass
ASM: Appendicular skeletal muscle mass
HDL-C: High-density lipoprotein cholesterol
LDL-C: Low-density lipoprotein cholesterol
TC: Total cholesterol
TGs: Triglycerides
95% CI: 95% Confidence intervals
AUC: Area under the curve
ROC: Receiver operating characteristic
